# Differential efficacy of subtalar fusion with three operative approaches

**DOI:** 10.1186/s13018-014-0115-2

**Published:** 2014-11-19

**Authors:** Cheng-song Yuan, Xiao-kang Tan, Bing-Hua Zhou, Jun-peng Liu, Xu Tao, Kang-Lai Tang

**Affiliations:** Department of Orthopaedics, Southwest Hospital, Third Military Medical University, No. 30, Gaotanyan Road, Chongqing, 400038 China

**Keywords:** Subtalar joint, Arthritis, Arthrodesis, Operative approach, Controlled clinical trial

## Abstract

**Background:**

There are many existing operative approaches for subtalar fusion; however, no optional strategy of operative approach has been developed yet. This study aimed to analyze the differential clinical efficacy of subtalar fusion with three operative approaches.

**Methods:**

The clinical data of 102 patients from April 2008 to April 2012 were analyzed prospectively. These patients were divided into three groups with the random number table: group A, group B, and group C. The following parameters were compared among three groups: effective exposure area and exposure time of subtalar joint, intraoperative bleeding volume, postoperative complications, fusion time, fusion rate, AOFAS score and VAS score before and after operation.

**Results:**

In the exposure area score, there was no statistically significant difference between group A and group C (*P* >0.05) ,but with a statistically significant difference between group A/C and group B (*P* <0.05). In exposure time and intraoperative bleeding volume, there was no statistically significant difference between group A and group B (*P* >0.05) but with a statistically significant difference between group A/B and group C (*P* <0.05). In three groups, there was a statistically significant difference in both AOFAS score and VAS score before operation and at 6 months/12 months/last visit after operation (*P* <0.05). The incidence of complications in the three groups was 8.8%, 12.5% and 19.4%. No statistically significant differences in fusion rate and fusion time were observed among the three groups (*P* >0.05).

**Conclusion:**

Three operative approaches have different indications, All the three operative approaches do not influence the fusion rate and fusion time of subtalar joint. The lateral tarsal sinus approach is inferior to the posterior-lateral L approach and the approach from the inferior tip of fibula to the basilar part of the fourth metatarsal bone in the exposure area, while the lateral tarsal sinus approach and the approach from the inferior tip of fibula to the basilar part of the fourth metatarsal bone are superior to the posterior-lateral L approach in the exposure time, intraoperative bleeding volume, and incidence of complications.

**Level of evidence:**

Therapeutic, level III.

## Background

Subtalar fusion is a gold standard for treatment of severe subtalar arthritis [1] and the operative factors affecting its efficacy include operative approach, cartilage removal, bone graft, hind foot deformity correction, and fixation [2]. Since subtalar joint has a highly complex anatomy and biomechanical characteristics, we presumed that the correct selection of operative approach might not only influence the trauma, prognosis, and occurrence of complications of patients but also play an important role in the operative field, operator’s operations, and good exposure.

At present, there are many approaches available for subtalar fusion, generally lateral, medial approaches [3]. The common lateral approaches include an approach from the inferior tip of fibula to the basilar part of the fourth metatarsal bone, a lateral tarsal sinus approach, and a lateral long-L approach. The subtalar joint is routinely accessed with a lateral approach [4]. Therefore, we only compared the clinical effects of different lateral approaches with a purpose of providing a basis for the selection of operative approach.

With the final purpose of reducing potential injury and postoperative complications and providing the best clinical selection strategy of operative approaches for subtalar fusion, this study aimed to explore the differential efficacy of subtalar fusion with different operative approaches and thus figure out the preferable clinical therapy through the prospective, comparative analysis on the clinical data of 102 patients who underwent subtalar fusion *in situ* with three operative approaches from April 2008 to April 2012.

## Materials and methods

### Study objects

This study was a prospective, randomized, single-blinded, controlled study and approved by the Ethics Committee of the Southwest Hospital. All included patients were assigned to groups A, B, and C according to the random number table. Before operation, X-ray examination, CT and MRI, as well as American Orthopaedic Foot and Ankle Society (AOFAS) scoring, VAS scoring, and admission evaluation were performed routinely.

#### Inclusion criteria

The inclusion criteria are as follows: (1) severe subtalar arthritis, i.e., peri-subtalar joint pain, aggravated when walking, seriously influencing the daily life; (2) failure of more than 6 months conservative treatment with drugs, local blockage, and physical rehabilitation; (3) fusion *in situ*; and (4) the patients and their family provided the informed consent.

#### Exclusion criteria

The exclusion criteria are as follows: (1) concomitant severe medical conditions, (2) severe subtalar joint infection, (3) severe osteoporosis, (4) hind foot deformity requiring orthopedic osteotomy, and (5) the patients and their family failed to provide the informed consent.

### Treatment

Group A: subtalar fusion *in situ* with an approach from the inferior tip of fibula to the basilar part of the fourth metatarsal bone, *n* = 34; group B: subtalar fusion *in situ* with a lateral tarsal sinus approach, *n* = 32; group C: subtalar fusion *in situ* with a posterior-lateral L approach, *n* = 36.

**Figure 1:**
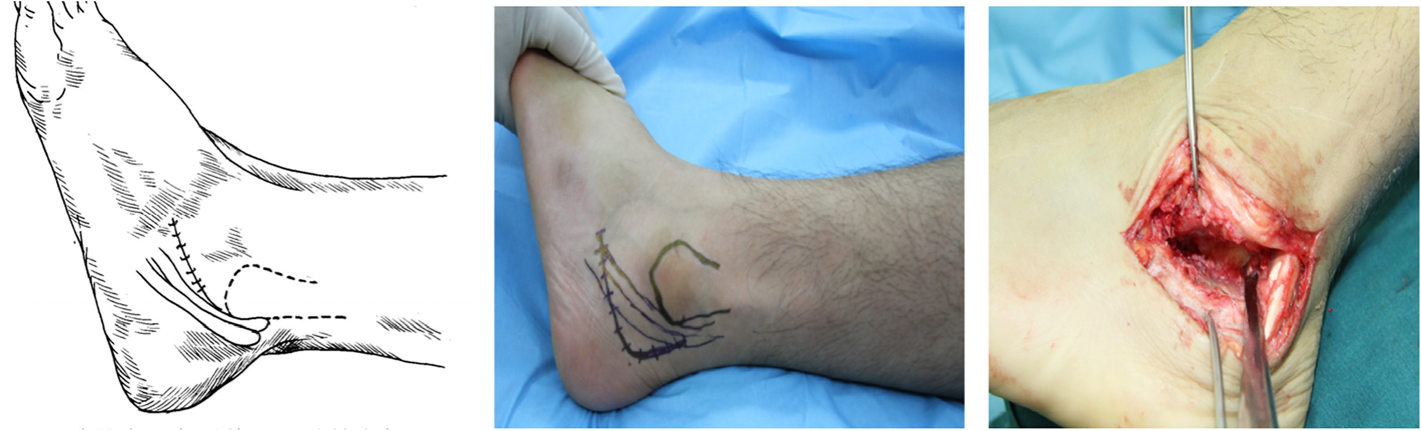
**An approach from the inferior tip of fibula to the basilar part of the fourth metatarsal bone and its exposure area.**

**Figure 2:**
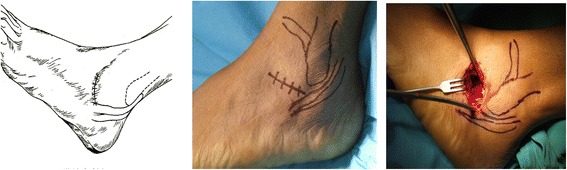
**A lateral tarsal sinus approach and its exposure area.**

**Figure 3:**
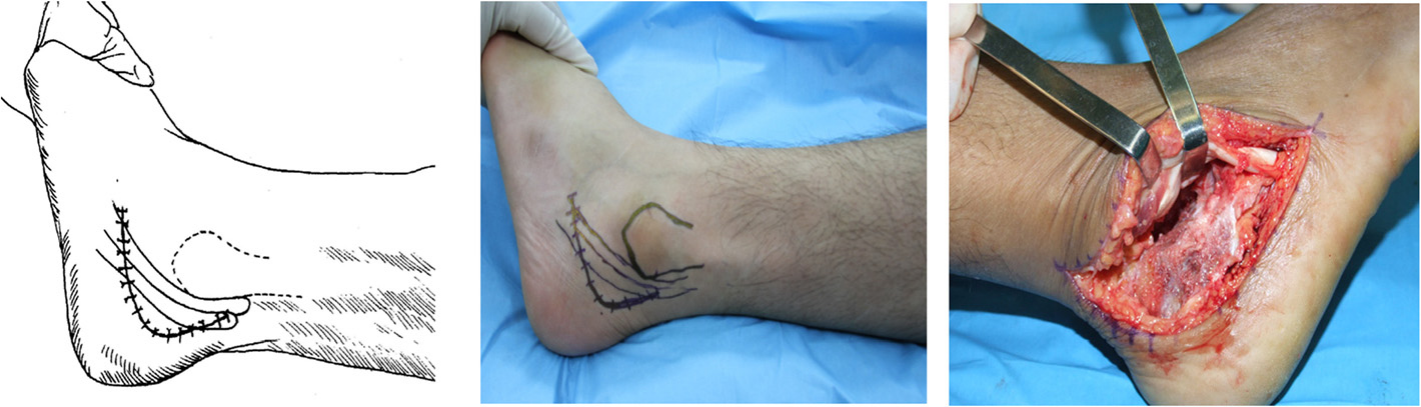
**A posterior-lateral L approach and its exposure area.**

The subtalar fusion in all included patients was performed by a chief physician with more than 15 years of clinical experience. The operative procedures were as follows: the antibiotic was intravenously infused for anti-infection 30 min before operation, and then lumbar anesthesia or general anesthesia was performed depending on the patient’s body condition. In all patients, the tourniquet (drive belt) was used at the root of the thigh for blood driving during operation, followed by conventional disinfection and draping. After the drive belt drove blood, the inflation pressure of tourniquet was 225 mmHg. Group A: when the tip of fibula was touched, a 3- to 5-cm incision parallel to subtalar articular facet was made extending to the basilar part of the fourth metatarsal bone at 0.5 cm beneath the tip of fibula (Figure 1); group B: when tarsal sinus was touched, a 2- to 4-cm short arc incision parallel to tarsal sinus was made with the center at 2 cm beneath lateral malleolus (Figure 2); group C: a 5- to 7-cm incision was made extending to the basilar part of the fifth metatarsal bone with the longitudinal approach at 2 to 3 cm over the lateral malleolus and along tendo calcaneus and the short and long muscle tendons of the fibula and with the horizontal approach between the tip of the lateral malleolus and the footplate skin (Figure 3). Thereafter, the cartilage on the articular facet was removed with the osteotome, bone file, and curette to expose 2 mm subchondral bone, and finally the subtalar joint was fixed by two 7.3-mm cannulated screws, one traversing the lateral third of the talar neck into the calcaneus and the other through the medial third of the talar neck into the calcaneus in two directions: plantar-to-dorsal (P-D) and dorsal-to-plantar (D-P). After operation, no leg plaster fixation was performed for the affected limb, but 3 to 5 days of continuous ice compress was performed and thereafter the ankle joint was subject to active and passive functional trainings. The suture was removed at 2 to 3 weeks postoperatively. No load was allowed within 6 weeks postoperatively, and thereafter partial load was allowed if callus was formed on the fusion surface as shown by X-ray reexamination after 6 weeks and a load was not allowed if no callus was formed. At 8 weeks postoperatively, partial load depended on whether there was callus formation on the fusion surface or not as shown by reexamination. At 3 months postoperatively, complete load was allowed if X-ray examination showed complete fusion of subtalar joint (i.e., the joint space disappeared completely and there was continuous callus passing through the fusion surface), and CT examination might be performed when the complete fusion of subtalar joint could not be evaluated by X-ray examination.

### Observations

#### Comparison of general data

The general data including sex, age, and course of disease before operation were compared among the three groups.

#### Comparison of intraoperative condition

The effective exposure area and exposure time of subtalar joint and intraoperative condition (bleeding volume and complications) were compared among the three groups. The following scoring criteria of subtalar joint exposure area during operation were used: the posterior subtalar articular facet was quartered and observed via the approaches; 10 scores: 4/4 exposure, 7 scores: 3/4 exposure, 5 scores: 2/4 exposure, 3 scores: 1/4 exposure, and 0 score: 0/4 exposure. The exposure time was recorded after the start of operation, i.e., time from skin incision to subtalar joint exposure (precision: 1 min). And the intraoperative bleeding volume was recorded with the planimetry (i.e., 1 cm^2^ blood-wetted gauze indicated 1 ml bleeding).

#### Comparison of hind foot pain and function

Before operation, at 6 months, 12 months, and the last visit after operation, the hind foot pain and function of all patients were evaluated and compared with the VAS score and AOFAS score.

#### Comparison of postoperative complications

Early complications included wound infection, skin necrosis, nerve injury, and articular facet dislocation, while late complications were articular facet non-union and adjacent joint osteoarthritis. The incidence of complications was compared among the three groups.

#### Comparison of postoperative fusion

At 6 weeks, 3 months, 6 months, 12 months, and the last visit after operation, X-ray examination was performed, including lateral, anterioposterior, and axial views of the calcaneus. The fusion time and fusion rate were compared among the three groups. The imaging-based fusion evaluation criterion was as follows: the radiographs and CT images showed that there was bone trabecula passing through the subtalar articular facet and the joint space disappeared completely; CT examination might be performed when the complete fusion of subtalar joint could not be evaluated by X-ray examination, because it was the gold standard to evaluate the fusion of subtalar joint.

### Statistical analysis

SPSS 13.0 software was used for statistical analysis of all data. The quantitative data were presented as mean ± standard deviation ($\overline{\chi}\pm s$) and compared with *t* test, while the qualitative data were compared with *X*^2^ test. *P* <0.05 suggested a statistically significant difference.

## Results

### Comparison of general data

Totally, 102 patients with severe subtalar arthritis were included in this study, including 56 cases of traumatic arthritis, 16 cases of rheumatoid arthritis, 17 cases of osteoarthritis, 10 cases of subtalar joint tuberculosis, and 3 cases of talocalcaneal coalition. There were 64 males and 58 females, with an age of 16 to 74 years (mean: 43.2 ± 3.6 years) and a course of disease before operation being 1 to 360 months (mean: 38.0 months). There were no statistically significant differences in general data among group A (*n* = 34), group B (*n* = 32), and group C (*n* = 36) (*P* >0.05), while the general data of the three groups were comparable, as shown in Table 1.

**Table 1:** **Comparison of general data among the three groups**

	**Number**	**Sex (M/F)**	**Age (year)**	**Course of disease (month)**	**Preoperative AOFAS score**	**Preoperative VAS score**	**Disease type**				
							**Traumatic arthritis (** ***n*** **)**	**Rheumatoid arthritis (** ***n*** **)**	**Osteoarthritis (** ***n*** **)**	**Tuberculosis (** ***n*** **)**	**Talocalcaneal coalition (** ***n*** **)**
Group A	34	23/11	39.4 ± 6.3	38.4 ± 5.9	50.0 ± 8.9	6.05 ± 1.3	18	7	4	3	2
Group B	32	19/13	44.9 ± 5.2	35.2 ± 5.8	47.0 ± 6.2	6.38 ± 1.6	21	5	3	0	3
Group C	36	22/14	45.3 ± 4.5	40.1 ± 6.9	49.9 ± 5.3	7.06 ± 1.8	15	4	10	5	2

*Abbreviations*: *M* male, *F* female.

*P* >0.05.

### Comparison of intraoperative condition

#### Comparison of effective exposure area

The average exposure area score of groups A, B, and C was 8.91, 6.56, and 9.16, respectively. The results of independent sample *t* test for comparison of average exposure area score showed that there was no statistically significant difference between group A and group C (*P* >0.05) but with a statistically significant difference between group A/C and group B (*P* <0.05) (see Table 2).

**Table 2:** **Comparison of exposure among three groups**

	**Number**	**4/4 exposure (** ***n*** **)**	**3/4 exposure (** ***n*** **)**	**2/4 exposure (** ***n*** **)**	**1/4 exposure (** ***n*** **)**	**Exposure score**
Group A	34	23	9	2	0	8.91
Group B	32	6	14	8	4	6.56
Group C	36	28	7	1	0	9.16

Compared with group B, *P* <0.05; when group A was compared with group C, *P* >0.05.

#### Comparison of exposure time and intraoperative bleeding volume

According to the results of independent sample *t* test for comparison of exposure time and intraoperative bleeding volume, there was no statistically significant difference between group A and group B (*P* >0.05) but with a statistically significant difference between group A/B and group C (*P* <0.05) (see Table 3).

**Table 3:** **Comparison of exposure time and intraoperative bleeding volume among the three groups**

	**Number**	**Average bleeding volume (ml)**	**Average exposure time (min)**
Group A	34	8.2 ± 2.3	3.2 ± 2.1
Group B	32	6.8 ± 1.2	2.8 ± 1.7
Group C	36	15.4 ± 1.3	3.6 ± 2.5

Compared with group C, *P* <0.05; when group A was compared with group B, *P* >0.05.

### Comparison of hind foot pain and function

There was a statistically significant difference before the operation and at 6 months/12 months/the last visit after operation (*P* <0.05). At 6 months, 12 months, and the last visit after operation, there were no statistically significant differences among the three groups (*P* >0.05).

### Comparison of postoperative complications

There were 14/102 patients who developed mild complications, with an incidence of 13.7%. In group A, one case experienced wound numbness, one case experienced anterior lateral foot numbness, and one case experienced hind foot pain; in group B, one case developed wound numbness, one case developed anterior lateral foot numbness, one case developed lateral foot dorsum numbness, and one case developed middle foot numbness; and in group C, two cases had wound numbness, three cases had anterior lateral foot numbness, one case had lateral foot dorsum numbness, and one case had toe numbness. The incidence of complications in the three groups was 8.8%, 12.5%, and 19.4%, respectively. For comparison of the incidence of complications among the three groups, the results of *t* test showed *P* <0.05, thus it was believed statistically that even with careful operation and enhanced care and under the premise of avoiding complications, the incidence of complications in group C was markedly higher than those in group A and group B.

### Comparison of postoperative fusion

**Figure 4:**
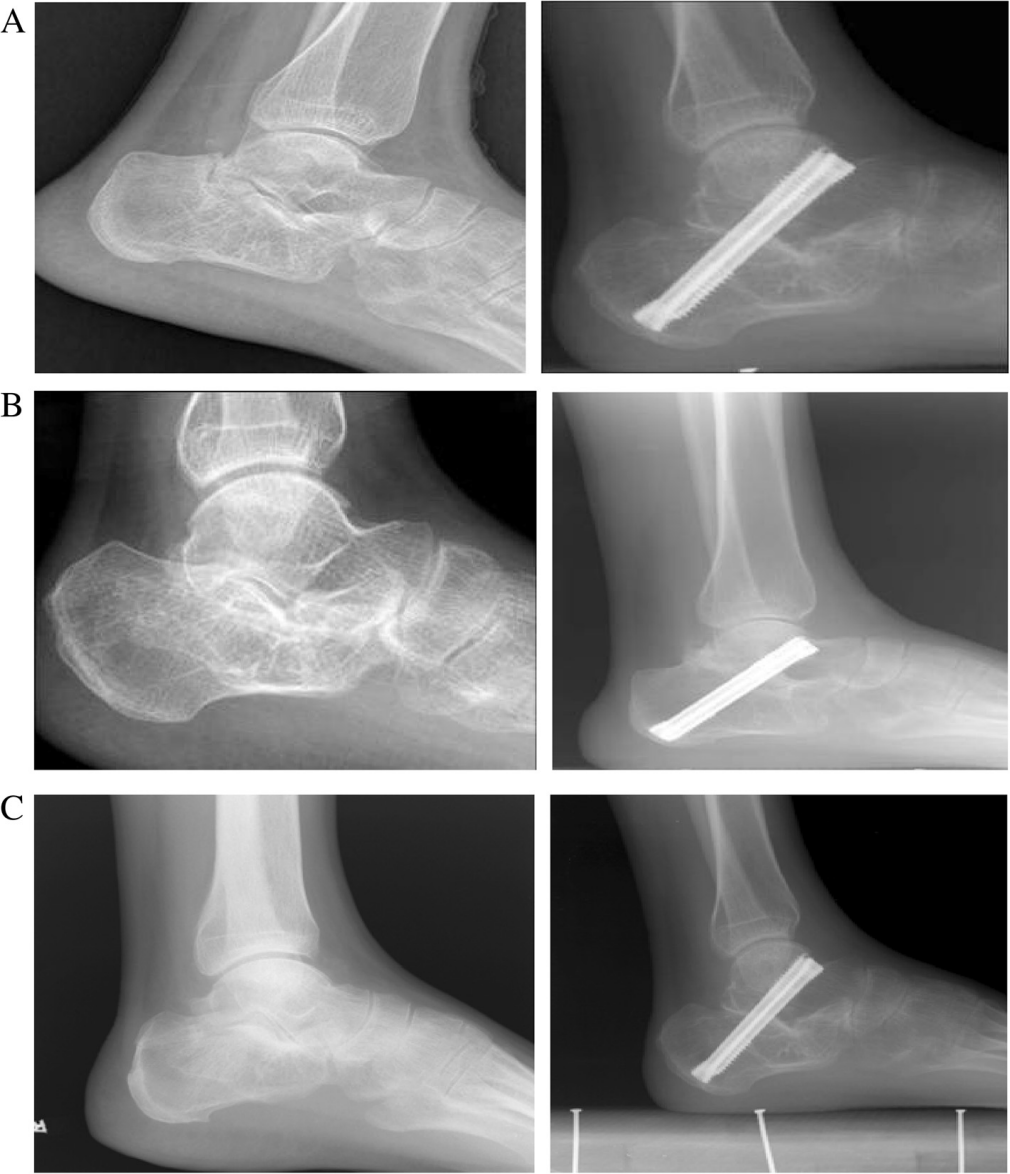
**Radiographs of the three groups before operation and at the last visit.** Group **A** (top), group **B** (center), and group **C** (bottom).

The radiographs confirmed that all cases except one case in the three groups achieved complete bone union (Figure 4); in the aspects of fusion time and fusion rate (Table 4), the results of *t* test showed no statistically significant differences among the three groups.

**Table 4:** **Comparison of fusion time and fusion rate among the three groups**

	**Number**	**Fusion time (weeks)**	**Fusion rate (%)**
Group A	34	9.6	97
Group B	32	10.2	100
Group C	36	9.8	100

*P* >0.05.

## Discussion

### Selection of operative approaches for subtalar fusion

Subtalar fusion is a common therapy for subtalar joint pain, instability or deformity with no response to conservative treatment [1], and its indications include traumatic subtalar arthritis, collapsed calcaneal fracture, flat foot, infectious arthritis, rheumatoid arthritis, subtalar joint tuberculosis, talocalcaneal coalition, neuromuscular disorders, and so on [4]. The surgical factors influencing the efficacy of subtalar fusion are operative approach, cartilage removal, bone graft, and fixation [3]. The selection of an applicable operative approach plays a critical role in clearly exposing subtalar joint, completely removing cartilages, effectively protecting nerves and blood vessels, and preventing complications [5]. Arthroscopic approach and open approach are most frequently used in clinical practice. Arthroscopic approach has many advantages, such as accurate localization, miniature invasion, clearer operative field [6], and capability to reduce the damage to soft tissues adjacent to the joint and thus provide good blood supply for joint fusion, which is the development direction of mini-invasive surgery [7]. However, this approach has limited indications and cannot effectively correct the cases of hind foot force line and joint space loss [8]. Therefore, open approach still has an irreplaceable role. Currently, there are various open approaches, including a lateral tarsal sinus approach, a lateral long-L approach, an approach from the inferior tip of fibula to the basilar part of the fourth metatarsal bone [3], as well as a posterior approach parallel to tendo calcaneus which was designed by Kalamchi and Evans [9]. Among these open approaches, a lateral tarsal sinus approach, an approach from the inferior tip of fibula to the basilar part of the fourth metatarsal bone, and a posterior-lateral long-L approach are used most commonly.

Three common approaches have their own advantages. A lateral tarsal sinus approach has such advantages as a small damage to adjacent soft tissues, simple exposure of subtalar joint, no requirement for cutting talocalcaneal ligaments and calcaneofibular ligaments during operation [10], and access to proper correction of hind foot deformity [5]. An approach from the inferior tip of fibula to the basilar part of the fourth metatarsal bone can directly expose subtalar joint, cause a small damage to adjacent soft tissues, avoid contacting sural nerve, greatly correct hind foot deformity, and simply remove the cartilage surface [11]. And a posterior-lateral L approach can provide extensive exposure, achieve good exposure of subtalar joint, and correct severe hind foot deformity [10].

According to our presumption and experience, the selection of operative approaches is based on the characteristics and severity of diseases and the operative purpose. The posterior-lateral L approach is a good choice for major bone grafting and extensive exposure; the lateral tarsal sinus approach and the approach from the inferior tip of fibula to the basilar part of the fourth metatarsal bone are both preferable for fusion *in situ* and low exposure, for example, a surgery for simple traumatic arthritis, talocalcaneal coalition, or rheumatoid arthritis often requires a low exposure area of subtalar joint due to no significant osteosclerosis, so the two approaches can be used; and in the cases of traumatic arthritis with collapsed calcaneal fracture, subtalar joint tuberculosis and osteoarthritis, the posterior-lateral L approach is the favorite of many surgeons, because it needs bone grafting, extensive and complete removal of affected tissues, and a high exposure area.

### Relation between operative approaches and complications

After subtalar fusion, the occurrence of foot dorsum numbness and anterior or middle lateral foot numbness is mainly associated with the injury of cutaneous nerves originating from sural nerve and superficial peroneal nerve in the lateral hind foot [12]. Sural nerve injury is often followed by skin sensation disappearance of lateral footplate border and superficial peroneal nerve injury by skin sensation disappearance of foot dorsum and anterior talocrural region [13]. The postoperative follow-up indicated the injury of both sural nerve and superficial peroneal nerve in five cases with a lateral tarsal sinus approach, seven cases with a posterior-lateral long-L approach, and two cases with an approach from the inferior tip of fibula to the basilar part of the fourth metatarsal bone.

In a review of relevant study results at home and aboard, Weinraub et al. [14] used the posterior-lateral long-L approach in the subtalar fusion of 28 patients and found that the fusion rate reached 100% and the fusion time was 8.5 (6 ~ 20) weeks, one patient experienced posterior lateral calcaneal pain, one patient experienced incision dehiscence, and one patient experienced anterior lateral foot numbness. Troy et al. [15] conducted subtalar fusion with the approach from the inferior tip of fibula to the basilar part of the fourth metatarsal bone in 31 patients, and the results showed that the fusion rate was 96% and the average fusion time was 10.6 weeks, one patient developed toe numbness and one patient developed lateral foot dorsum numbness. Pollard et al. [16] performed subtalar fusion using the posterior-lateral long-L approach in 22 patients and finished a follow-up of 27.3 (12 ~ 63.9) months, and they found that the fusion rate was 95.5%, one case developed bone non-union, three cases developed wound dehiscence, three cases developed wound numbness, and seven cases developed metal-response pain. In a study of subtalar fusion with the posterior-lateral long-L approach in 15 patients, Lee et al. [17] found the fusion rate was 93.3%, two patients had anterior lateral foot numbness due to sural nerve injury and two patients had wound dehiscence. Therefore, from either the anatomy or the clinical follow-up results, the incidence of the nerve injury with the posterior-lateral L approach and the lateral tarsal sinus approach is higher than that with the approach from the inferior tip of fibula to the basilar part of the fourth metatarsal bone.

## Conclusion

Three operative approaches have different indications, thus an applicable approach shall be selected according to the disease characteristics and the operative purpose. All the three operative approaches do not influence the fusion rate and fusion time of subtalar joint. The lateral tarsal sinus approach is inferior to the posterior-lateral L approach and the approach from the inferior tip of fibula to the basilar part of the fourth metatarsal bone in exposure area, while the lateral tarsal sinus approach and the approach from the inferior tip of fibula to the basilar part of the fourth metatarsal bone are superior to the posterior-lateral L approach in exposure time, intraoperative bleeding volume, and incidence of complications.
